# The development trajectory of self-advocacy in patients with adjuvant chemotherapy after breast cancer surgery and its predictors

**DOI:** 10.1097/MD.0000000000049197

**Published:** 2026-06-05

**Authors:** Shuyuan Lei, Songyan Zhao, Xi Chen

**Affiliations:** a School of Nursing, Inner Mongolia Medical University, Hohhot, Inner Mongolia, China; b Chifeng Municipal Hospital, Chifeng, Inner Mongolia, China.

**Keywords:** adjuvant chemotherapy, breast cancer, predictors, self-advocacy

## Abstract

There is still a lack of research to analyze the development trajectory of the level of self-advocacy and related factors in patients with adjuvant chemotherapy after breast cancer. The present study aims to analyze the development trajectory of self-advocacy and its predictors in patients with adjuvant chemotherapy after breast cancer. A total of 242 breast cancer patients admitted to our hospital from January 2024 to December 2024 were collected. A total of 200 patients participated in 4 follow-up surveys before chemotherapy (T1), early chemotherapy (T2), mid-chemotherapy (T3), and late chemotherapy (T4), with a valid questionnaire recovery rate of 82.64%. Latent class growth analysis was used to analyze the development trajectory of self-advocacy in patients with postoperative adjuvant chemotherapy for breast cancer, and the influencing factors’ development trajectory of self-advocacy level was analyzed by binary logistic regression. The self-advocacy scores of breast cancer patients with adjuvant chemotherapy after surgery at T1 to T4 were (56.49 ± 13.89), (56.69 ± 14.50), (57.15 ± 16.89), and (56.59 ± 19.96) points, respectively. The scores of self-advocacy at the 4 time points were positively correlated (*P* < .05), which met the premise of model fitting. The development trajectory of self-advocacy of patients with adjuvant chemotherapy after breast cancer surgery was plotted, and the patients were divided into high self-advocacy group (n = 85) and low self-advocacy group (n = 115) according to the development trajectory. Multivariate logistic regression analysis showed that breast-conserving surgery, education, Consumer Experiences of Stigma Questionnaire score, and Nurse Patient Trust Scale score were the influencing factors of the development trajectory of self-advocacy in patients with adjuvant chemotherapy after breast cancer surgery (*P* < .05). Breast-conserving surgery, education, Consumer Experiences of Stigma Questionnaire score, and Nurse Patient Trust Scale score were the influencing factors of the development trajectory of self-advocacy in patients with adjuvant chemotherapy after breast cancer surgery.

## 1. Introduction

Self-advocacy has a positive effect on patients that cannot be ignored. A previous study found that through self-advocacy, patients with mental disorders could actively acquire health knowledge and skills and adopt health-promoting behaviors, which can not only effectively control disease symptoms but also enhance patients’ sense of self-identity and increase their participation in social activities.^[1]^ It is worth emphasizing that the potential value of self-advocacy among cancer survivors is not limited to this. Patients with a high level of self-advocacy can make informed decisions based on their health status and proactively address health issues through communication with healthcare providers, thereby reducing emergency department visits and hospitalizations.^[2–4]^ Self-advocacy also enhances cancer survivors’ sense of responsibility for their own care, thereby improving the quality of self-management, reducing symptoms, and improving well-being. Hagan and Donovan conducted a mixed study of 179 female cancer survivors and found that high self-advocacy was beneficial for reducing symptom severity.^[5]^ In a subsequent study in 2016, Hagan, Cohen, Stone, and others developed the Female Self-Advocacy In Cancer Survivorship Scale (FSACS) for female cancer survivors.^[6]^ Then, the good reliability and validity of FSACS were verified in another study.^[7]^

Surgical treatment of breast cancer can disrupt the integrity of a woman’s secondary sexual characteristics, causing patients to experience disturbed self-image, gender identity disorders, and even a strong sense of stigma.^[8–10]^ Patient–nurse trust is theoretically defined as confidence in appropriate, reliable, and successful nursing care. In clinical practice, nurses and patients are participants in patient–nurse trust, and establishing a strong trust relationship can help nurses establish close cooperation with patients, enable nurses to take comprehensive care of patients, promote continuous care and treatment, improve patient compliance with treatment and care, and further promote patient recovery.^[11]^ Both stigma and patient–nurse trust may have an impact on patients’ level of self-advocacy, but there is currently a lack of relevant research. The present study aims to analyze the development trajectory of self-advocacy in patients undergoing adjuvant chemotherapy after breast cancer surgery and its predictors.

## 2. Information and methods

### 2.1. General information

A total of 242 breast cancer patients admitted to our hospital from January 2024 to December 2024 were collected. A total of 200 patients participated in 4 follow-up surveys before chemotherapy (T1), early chemotherapy (T2), mid-chemotherapy (T3), and late chemotherapy (T4), with a valid questionnaire recovery rate of 82.64%. Inclusion criteria were the following: pathological diagnosis of breast cancer; preparing to receive adjuvant chemotherapy after breast cancer surgery; age ≥ 18 years old, female patients; be able to communicate and exchange effectively; and informed consent has been obtained and voluntarily participated in the present study. Exclusion criteria are as follows: patients who received neoadjuvant chemotherapy before surgery; tumor recurrence or distant metastasis (metastasis to other organs, such as lung, liver, and brain; or metastasis to other tissues, such as non-regional lymph nodes and bones); cognitive dysfunction; history of mental illness; serious diseases of important organs such as heart, brain, lungs, liver, and kidneys; and complications such as severe systemic infection, severe anemia. Standard for detachment: concurrent chemoradiotherapy or endocrinotherapy; chemotherapy was terminated for various reasons during the investigation period; the assessment cannot be completed during the survey.

### 2.2. Ethics statement

**Figure 1.**
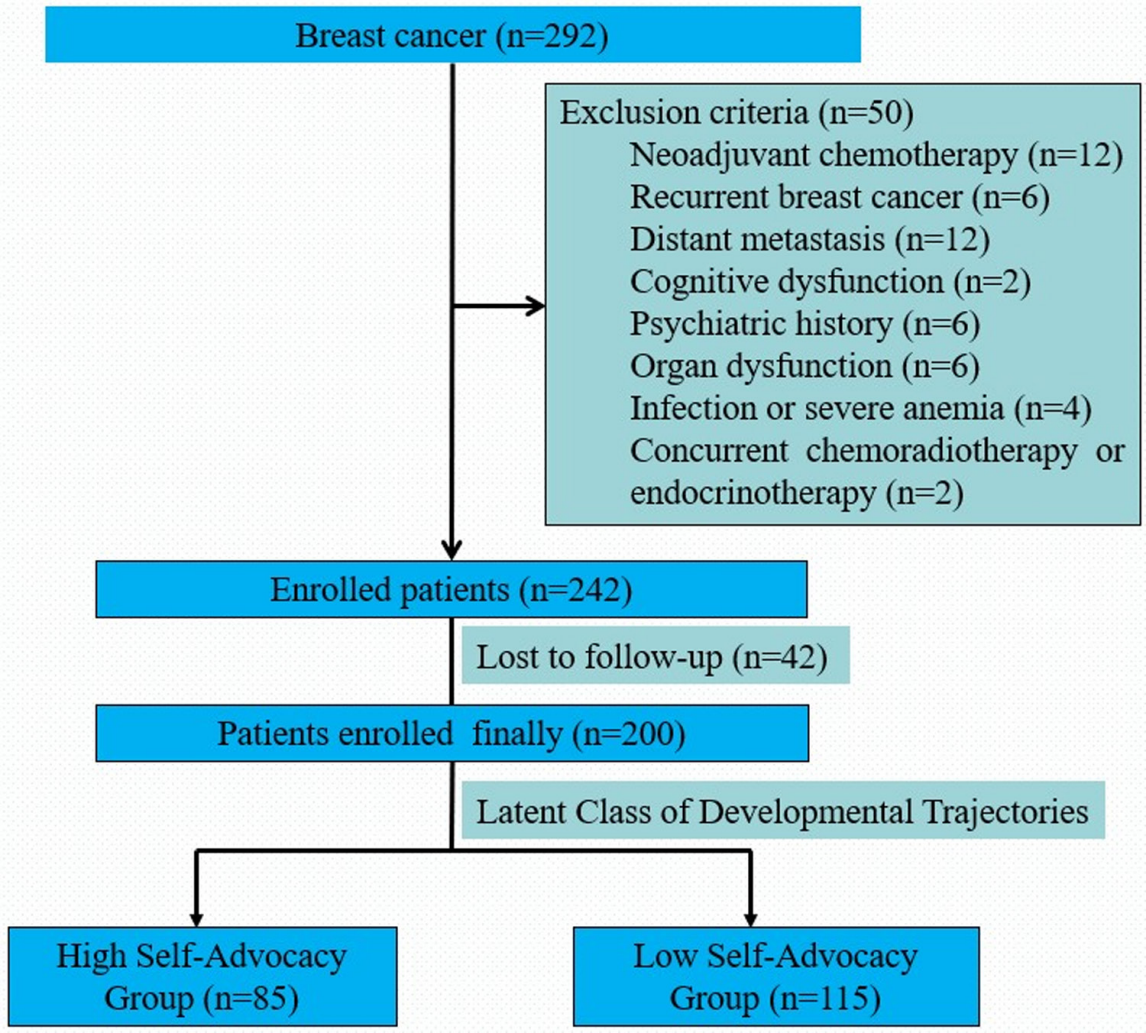
Flow chart of patient inclusion.

The present study was approved by the ethics committee of our hospital (No. CK20250111), and all patients had signed the informed consent form. The flow chart of patient inclusion can be seen in Figure 1.

### 2.3. Survey tools

#### 2.3.1. General information

The age, place of residence, marital status, work status, annual family income, medical insurance type, number of children, primary caregivers, breast cancer site, smoking history, alcohol abuse history, body mass index, family history, menstrual status, hypertension, diabetes, hyperlipidemia, tumor size, Human Epidermal Growth Factor Receptor 2, Ki-67, estrogen receptor, and progesterone receptor expression level, luminal type, lymph node metastasis, chemotherapy cycle, education level, and surgical method were collected.

#### 2.3.2. FSACS score scale

FSACS was used to evaluate the level of self-advocacy, including 3 dimensions: self-decision-making (6 items), effective communication (6 items), and social support (6 items).

#### 2.3.3. Consumer Experiences of Stigma Questionnaire

The Consumer Experiences of Stigma Questionnaire (CESQ) questionnaire consisted of 9 items, including the stigma generated during interpersonal communication and the experience of discrimination, and was scored on a 5-point scale, with higher scores indicating that the patient’s actual stigma was stronger. The CESQ was measured at baseline only.

#### 2.3.4. Nurse Patient Trust Scale

The Nurse Patient Trust Scale (NPTS) consisted of 2 dimensions, including 6 items for attitude and care, and 6 items for ability and sense of security, using the Likert 4-level scoring method. Higher scores indicated higher levels of patient trust in nurses. The NPTS was measured at baseline only.

### 2.4. Quality control

The data were checked and entered by 2 independent researchers, and the valid questionnaires were numbered and entered immediately after elimination. For inconsistent data, the researcher would correct the data after verifying it and invited statistical experts to guide the data analysis method in the study and make modifications.

### 2.5. Statistical analysis

SPSS 26.0 (IBM Corporation, Armonk) and Mplus 8.3 (Muthén & Muthén, Los Angeles) were used to complete the data analysis of this study, and *P* < .05 indicated that the difference was statistically significant. The mean ± standard deviation of the mean data was used, and the independent-sample *t* test analyzed the differences between groups. The counting data were expressed as n (%), and the chi-square test was used to analyze the differences between groups. Multivariate binary logistic regression analysis analyzed the influencing factors of the development trajectory of self-advocacy. The latent class growth analysis was used to analyze the development trajectory of self-advocacy in patients with adjuvant chemotherapy after breast cancer surgery. Samples with missing data had been excluded. According to the requirements of multiple regression analysis, one independent variable corresponds to at least 10 samples. Four independent variables were planned to be included in the pilot experiment, and the number of cases in the present study met the research needs.

## 3. Results

### 3.1. The latent class growth analysis of self-advocacy level and development trajectory in patients with adjuvant chemotherapy after breast cancer

The self-advocacy scores of patients with adjuvant chemotherapy after breast cancer at T1 to T4 were (56.49 ± 13.89), (56.69 ± 14.50), (57.15 ± 16.89), and (56.59 ± 19.96), respectively, and the self-advocacy scores were relatively stable at different times. The scores of self-advocacy at the 4 time points were positively correlated, as shown in Table 1, which satisfied the premise of model fitting. Based on the 4 FSACS scores of patients as observation indicators, the final data of 200 patients were included and analyzed, and the latent class growth analysis of 1 to 5 categories was gradually established to identify the potential categories of the development trajectory of self-advocacy in patients with adjuvant chemotherapy after breast cancer. The results showed that with the increase in the number of fitting categories, the absolute values of the Akaike information criterion, Bayesian information criterion, and adjusted Bayesian information criterion of models 1 to 5 gradually decreased, and the Lo–Mendell–Rubin and Bootstrap Likelihood Ratio Test of models 1 to 4 were statistically significant (*P* < .05), while the Lo–Mendell–Rubin of model 5 was not statistically significant (*P* > .05). In addition, the classification results of model 3 and model 4 had a sample size of <5%.^[12]^ Therefore, model 2 was selected as the best fitting result, as shown in Table 2.

**Table 1:** Scores and correlation analysis of self-advocacy of patients undergoing adjuvant chemotherapy after breast cancer surgery (n = 200).

Model	T1	T2	T3	T4
T1	1	–	–	–
T2	0.767*	1	–	–
T3	0.752*	0.827*	1	–
T4	0.770*	0.809*	0.886*	1

* *P*** **<** **.001.

**Table 2:** Results of latent class growth analysis of development trajectory of self-advocacy in adjuvant chemotherapy patients after breast cancer surgery (n = 200).

Model	AIC	BIC	aBIC	Entropy value	*P* value		Category probability(%)
					LMR	BLRT	
1	6728.793	6748.583	6729.575	–	–	–	–
**2**	**5792.009**	**5821.693**	**5793.180**	**0.991**	**<.001**	**<.001**	**57.70/42.30**
3	5737.245	5776.825	5738.807	0.985	.003	<.001	56.50/39.00/0.045
4	5700.907	5750.382	5702.860	0.988	<.001	<.001	56.50/37.50/0.045/0.015
5	5687.298	5746.668	5689.642	0.921	.247	<.001	56.50/19.00/18.50/0.045/0.015

Bold values denote the best-fitting model (Model 2).

AIC = Akaike information criterion, aBIC = adjusted Bayesian information criterion, BIC = Bayesian information criterion, BLRT = Bootstrap Likelihood Ratio Test, LMR = Lo–Mendell–Rubin.

**Figure 2.**
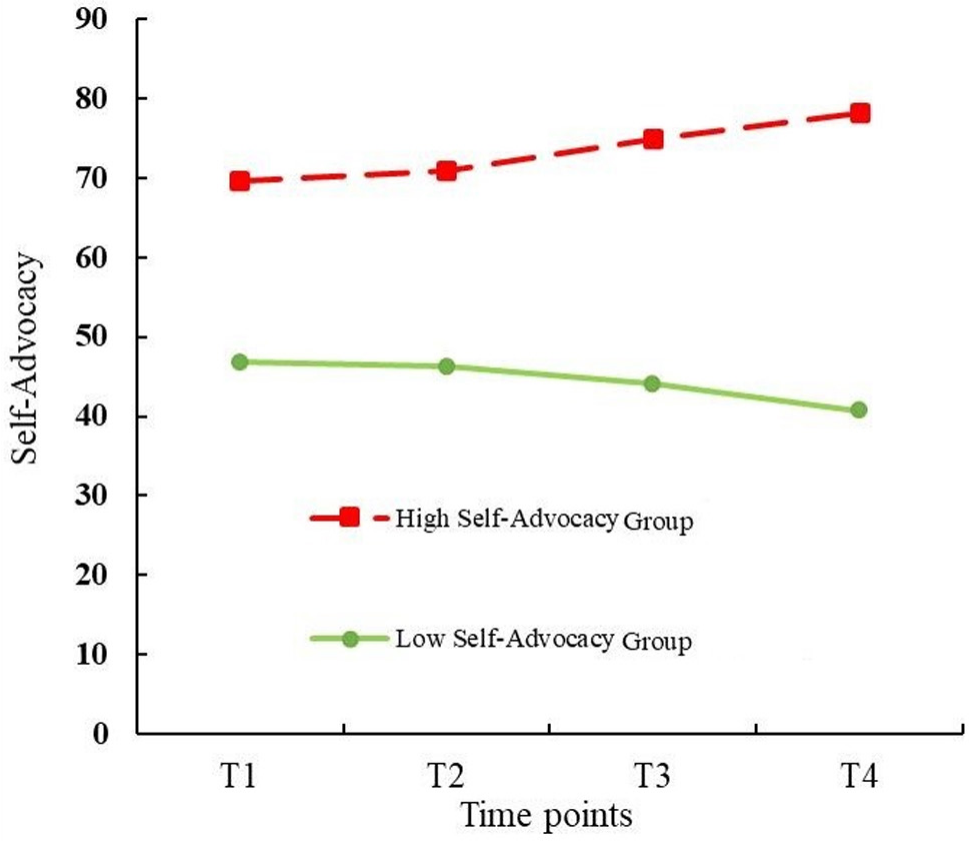
Categories of self-advocacy trajectories of patients with adjuvant chemotherapy after breast cancer surgery.

According to model 2, the development trajectory of self-advocacy in patients with adjuvant chemotherapy after breast cancer surgery was plotted, as shown in Figure 2. According to the development trajectory characteristics of the patients’ self-advocacy scores at different time points corresponding to the 2 development trajectories, they were named respectively: category 1: the initial level of self-advocacy of the patients in this subgroup was higher (I = 68.936, *P* < .001). Then, it gradually increased over time (S = 3.021, *P* < .001), named the “High Self-Advocacy Group,” accounting for 42.50% (n = 85) of the sample. Category 2: patients in this subgroup had a low initial level of self-advocacy (I = 47.619, *P* < .001), and then gradually decreased over time (S = −2.099, *P* < .001), named the “Low Self-Advocacy Group,” accounting for 57.50% of the sample (n = 115).

### 3.2. The main clinical characteristics of the survey subjects

Univariate analysis showed that there were significant differences in terms of education level, smoking history, history of alcoholism, Human Epidermal Growth Factor Receptor 2 positivity, breast-conserving surgery, luminal type, tumor size (X), CESQ score, and NPTS score (*P* < .05) between the 2 groups, as shown in Table 3.

**Table 3:** Univariate analysis of the general information.

Variables	Low self-advocacy group (n = 115)	High self-advocacy group (n = 85)	*t/*χ^2^	*P*
Age	48.15 ± 10.80	50.22 ± 10.37	1.366	.173
Place of residence			1.274	.259
Countryside	32 (27.8)	30 (35.3)		
City	83 (72.2)	55 (64.7)		
Education			22.316	<.001
High school or below	83 (72.2)	33 (38.8)		
Bachelor’s degree or above	32 (27.8)	52 (61.2)		
Marital status			0.900	.343
Married	22 (19.1)	21 (24.7)		
Others	93 (80.9)	64 (75.3)		
Occupational status			0.004	.951
Incumbency	78 (67.8)	58 (68.2)		
Dimission	37 (32.2)	27 (31.8)		
Annual family income			1.889	.169
<200,000 RMB	60 (52.2)	36 (42.4)		
≥200,000 RMB	55 (47.8)	49 (57.6)		
Medical insurance methods			0.071	.789
Medical insurance payment	93 (80.9)	70 (82.4)		
At one’s own expense	22 (19.1)	15 (17.6)		
Number of children			1.484	.476
0	36 (31.3)	20 (23.5)		
1	41 (35.7)	33 (38.8)		
≥2	38 (33.0)	32 (37.6)		
Primary caregiver			0.762	.383
Partner	68 (59.1)	45 (52.9)		
Others	47 (40.9)	40 (47.1)		
Body mass index (kg/m^2^)			2.108	.147
≤24	62 (53.9)	37 (43.5)		
>24	53 (46.1)	48 (56.5)		
Smoking history			6.057	.014
No	105 (91.3)	85 (100.0)		
Yes	10 (8.7)	0 (0.0)		
History of alcoholism			3.968	.046
No	105 (91.3)	84 (98.8)		
Yes	10 (8.7)	1 (1.2)		
Family history			-	.638
No	112 (97.4)	84 (98.8)		
Yes	3 (2.6)	1 (1.2)		
Menopause			3.301	.069
No	69 (60.0)	40 (47.1)		
Yes	46 (40.0)	45 (52.5)		
Hypertension			0.158	.691
No	106 (92.2)	77 (90.6)		
Yes	9 (7.8)	8 (9.4)		
Diabetes			0.636	.425
No	112 (97.4)	81 (95.3)		
Yes	3 (2.6)	4 (4.7)		
Hyperlipidemia			0.732	.392
No	109 (94.8)	78 (91.8)		
Yes	6 (5.2)	7 (8.2)		
Tumor site			0.852	.356
Left	56 (48.7)	47 (55.3)		
Right	59 (51.3)	38 (44.7)		
Positive HER-2			14.620	<.001
No	97 (84.3)	85 (100.0)		
Yes	18 (15.7)	0 (0.0)		
Ki67 expression			2.789	.095
Low	21 (18.3)	24 (28.2)		
High	94 (81.7)	61 (71.8)		
Estrogen receptor			0.422	.516
Negative	26 (22.6)	16 (18.8)		
Positive	89 (77.4)	69 (81.2)		
Progesterone receptor			0.043	.836
Negative	23 (20.0)	16 (18.8)		
Positive	92 (80.0)	69 (81.2)		
Luminal type			12.591	.006
Luminal A	19 (16.5)	22 (25.9)		
Luminal B	71 (61.7)	48 (56.5)		
HER-2 positive	12 (10.4)	0 (0.0)		
Triple negative	13 (11.3)	15 (17.6)		
Lymph node positive			1.466	.226
No	79 (68.7)	65 (76.5)		
Yes	36 (31.3)	20 (23.5)		
Breast-conserving surgery			31.879	<.001
No	101 (87.8)	44 (51.8)		
Yes	14 (12.2)	41 (48.2)		
Chemotherapy cycles			0.137	.934
4	34 (29.6)	27 (31.8)		
6	52 (45.2)	38 (44.7)		
8	29 (25.2)	20 (23.5)		
Tumor size (X)	2.21 ± 1.25	1.84 ± 1.03	2.275	.024
Tumor size (Y)	1.72 ± 0.99	1.47 ± 0.84	1.918	.057
Tumor size (Z)	12.8 ± 0.69	1.16 ± 0.56	1.297	.196
CESQ	31.23 ± 7.30	21.39 ± 5.56	15.598	<.001
NPTS	25.05 ± 5.90	36.68 ± 3.68	17.108	<.001

CESQ = Consumer Experiences of Stigma Questionnaire, HER-2 = Human Epidermal Growth Factor Receptor 2, NPTS = Nurse Patient Trust Scale.

### 3.3. Influencing factors of the development trajectory of self-advocacy in patients with adjuvant chemotherapy after breast cancer surgery

Statistically significant variables in univariate analysis were taken as independent variables, and the development trajectory of self-advocacy in patients with postoperative adjuvant chemotherapy for breast cancer was used as the dependent variable (low self-advocacy group = 0, high self-advocacy group = 1). The binary logistic regression analysis was conducted, and the independent variables are presented in Table 4. The results showed that breast-conserving surgery, education, CESQ score, and NPTS score were the influencing factors of the development trajectory of self-advocacy in patients with adjuvant chemotherapy after breast cancer surgery, and the differences were statistically significant (*P* < .05; Table 5).

**Table 4:** Assignment methods of independent variables.

Variables	Assignment method
Education	High school or below = 0; bachelor’s degree or above = 1
Smoking history	No = 0, yes = 1
History of alcoholism	No = 0, yes = 1
HER-2 positive	No = 0, yes = 1
Breast-conserving surgery	No = 0, yes = 1
Luminal type	Luminal A = 0; luminal B = 1; HER-2 positive = 2; triple negative = 3
Tumor size (X)	Original value
CESQ	Original value
NPTS	Original value

CESQ = Consumer Experiences of Stigma Questionnaire, HER-2 = Human Epidermal Growth Factor Receptor 2, NPTS = Nurse Patient Trust Scale.

**Table 5:** Binary logistic regression analysis of the development trajectory of self-advocacy in patients with adjuvant chemotherapy after breast cancer surgery (n = 200).

Variables	*Β* value	SE value	Wald χ^2^ value	*P* value	OR value	95% CI
Education	1.080	0.532	4.119	.042	2.943	1.038–8.349
Breast-conserving surgery	1.078	0.529	4.148	.042	2.939	1.041–8.293
CESQ	−0.140	0.040	12.581	<.001	0.869	0.804–0.939
NPTS	0.244	0.047	27.067	<.001	1.276	1.164–1.399
Constant	−5.394	1.899	8.066	.005	0.005	–

CESQ = Consumer Experiences of Stigma Questionnaire, CI = confidence interval, NPTS = Nurse Patient Trust Scale, OR = odds ratio, SE = standard error.

## 4. Discussion

Chemotherapy drugs are mainly based on the characteristics of tumor cells proliferating faster than normal cells and achieve the purpose of killing tumor cells by directly destroying the structure of tumor cells or blocking the substances required in the cell proliferation process. Chemotherapy is an important means of systemic treatment for breast cancer.^[13,14]^ However, chemotherapy can cause various adverse reactions, such as liver function damage, gastrointestinal reactions, allergies, hair loss, etc., resulting in a decrease in the quality of life of patients, causing a huge impact on the patient’s physical and mental state, and seriously affecting the clinical treatment effect.^[15,16]^ Self-advocacy can help patients seek help and make appropriate decisions,^[17]^ improve the quality of self-management of breast cancer patients during postoperative chemotherapy, reduce adverse reactions, and improve the efficacy of chemotherapy. The present study examined the self-advocacy level of patients with postoperative adjuvant chemotherapy for breast cancer at different time points and found that the level of self-advocacy at different time points in patients with adjuvant chemotherapy after breast surgery was at a moderate level, and 57.50% (115/200) of the patients belonged to the low self-advocacy decreasing group. In view of this characteristic, nursing staff can establish a good nurse–patient trust relationship and promote the development of patients’ self-advocacy level.^[18]^

To promote the level of self-advocacy in patients with adjuvant chemotherapy after breast cancer surgery, it was found that breast-conserving surgery, education, CESQ score, and NPTS score were independent influencing factors of the development trajectory of self-advocacy (*P* < .05). The present study found that breast-conserving surgery was positively correlated with the level of self-advocacy, which can allow women to better adapt to life after surgery, reduce psychological pressure and inferiority complex, and reduce patients’ anxiety and depression levels.^[19]^ A study in diabetic patients confirmed that the level of anxiety and depression was significantly negatively correlated with the level of self-advocacy.^[1]^ This indicated that breast-conserving surgery mainly aimed to reduce patients’ anxiety and depression, and then improve their self-advocacy level. Therefore, the rate of breast-conserving surgery should be actively elevated; then, the mental health status and self-advocacy level of patients could be improved. The higher the level of education, the stronger the ability to actively learn about breast cancer and chemotherapy, including possible symptoms and coping methods, and to communicate with medical staff more effectively and take effective countermeasures based on these problems.^[20]^ This is beneficial for improving patients’ confidence in overcoming the disease, reducing shame, and thus improving the self-advocacy level. In addition, the present study found that CESQ was negatively correlated with self-advocacy. The greater the stigma, the lower the level of self-advocacy. Stigma is the sense of shame or discrimination caused by illness (especially mental illness, infectious diseases, etc.), which mainly stems from social prejudice, misperception, or cultural concepts. Stigma can lead patients to conceal their condition, resist treatment, and even cause psychological problems, which is one of the important factors affecting disease recovery.^[21–23]^ A study in older female breast cancer survivors confirmed that stigma was inversely correlated with the level of self-advocacy.^[17]^ A study in patients with hereditary hemorrhagic telangiectasia also confirmed that stigma was negatively correlated with the level of self-advocacy.^[24]^ Finally, the present study found that the NPTS score was positively correlated with the level of self-advocacy. The nurse–patient relationship is the working and professional interpersonal relationship established by nursing staff with patients and their families, companions, guardians, and patients’ units in the medical process, involving caregivers, nurses, head nurses, and directors of the nursing department, throughout the whole process of patient treatment to discharge.^[25–27]^ The nurse–patient relationship aims to solve the physical and psychological needs of patients, including basic elements such as respect, equality, and empathetic communication.^[28,29]^ Establishing a good nurse–patient relationship can help nurses establish close cooperation with patients, promote the continuous improvement of nursing quality, improve patients’ compliance with nursing treatment, enhance patients’ sense of security, reduce stress, and be more willing to share their ideas with nurses and improve the level of self-advocacy.^[30,31]^

### 4.1. Limitations of the present study

This study was a retrospective single-center clinical study, and the regional limitations of the sample source limit the external applicability of the results of this study. Moreover, due to the limited sample size included in the present study, we did not include all variables in the multivariate regression analysis. The CESQ and NPTS scores were measured at baseline only.

### 4.2. Conclusion

Breast-conserving surgery, education, CESQ, and NPTS were the influencing factors in the development trajectory of self-advocacy in patients undergoing adjuvant chemotherapy after breast cancer surgery.

## Author contributions

**Conceptualization:** Shuyuan Lei, Songyan Zhao, Xi Chen.

**Data curation:** Shuyuan Lei, Songyan Zhao, Xi Chen.

**Investigation:** Shuyuan Lei, Songyan Zhao, Xi Chen.

**Methodology:** Shuyuan Lei, Songyan Zhao, Xi Chen.

**Supervision:** Songyan Zhao, Xi Chen.

**Writing – original draft:** Shuyuan Lei.

**Writing – review & editing:** Shuyuan Lei, Songyan Zhao, Xi Chen.

## References

[1] Xi N Wei C Chu Q. Mediating role of self-advocacy in the correlation between depression and health-related quality of life among elderly patients with diabetes mellitus. Panminerva Med. 2024;66:332–4. 37712863 10.23736/S0031-0808.23.04963-7

[2] Habibpour E Ramazanzadeh N Gardashkhani S Shahmari M. Psychometric properties of the Persian Self-Advocacy in Cancer Survivorship Scale (SACS-P) in Iran. Cancer Med. 2025;14:e71318. 41163344 10.1002/cam4.71318 PMC12571988

[3] Li Y Cheng L Xie Y Wang L Liang G Lou Y. Self-advocacy experiences in coping with dysphagia among patients with oral cancer: a qualitative study. Asia Pac J Oncol Nurs. 2025;12:100773. 40951451 10.1016/j.apjon.2025.100773 PMC12424248

[4] Habibpour E Shahmari M Ramazanzadeh N Gardashkhani S. Psychometric evaluation of self-advocacy scale in patients with cancer: a psychometric evaluation protocol in Iran. BMJ Open. 2025;15:e101888. 10.1136/bmjopen-2025-101888 PMC12265829 40664411

[5] Hagan TL Donovan HS. Ovarian cancer survivors’ experiences of self-advocacy: a focus group study. Oncol Nurs Forum. 2013;40:140–7. 23454476 10.1188/13.ONF.A12-A19 PMC4021021

[6] Hagan TL Cohen S Stone C Donovan H. Theoretical to tangible: creating a measure of self-advocacy for female cancer survivors. J Nurs Meas. 2016;24:428–41. 28714448 10.1891/1061-3749.24.3.428 PMC5514617

[7] Hagan TL Cohen SM Rosenzweig MQ Zorn K Stone CA Donovan HS. The Female Self-Advocacy in Cancer Survivorship Scale: a validation study. J Adv Nurs. 2018;74:976–87. 29117439 10.1111/jan.13498 PMC5844819

[8] Zoromba MA Deibes HM Abdelhady DH. Exploring breast cancer stigma among medical students in Egypt: a national multi-center cross-sectional study. BMC Public Health. 2025;25:3604. 41146145 10.1186/s12889-025-24656-2 PMC12557924

[9] Yan W Zhou H Huang Y Zhang Y Wang S Yu X. Factors influencing stigma in Chinese postoperative breast cancer patients: a systematic review and meta-analysis. Front Med (Lausanne). 2025;12:1681487. 41140688 10.3389/fmed.2025.1681487 PMC12546193

[10] Saleh MN Owuor K Helova A. Pilot study assessing stigma in Kenyan women with breast cancer. JCO Glob Oncol. 2025;11:e2400479. 41124624 10.1200/GO-24-00479

[11] Lubasch JS Lee S Kowalski C Beckmann M Pfaff H Ansmann L. Hospital processes and the nurse-patient interaction in breast cancer care. Findings from a cross-sectional study. Int J Environ Res Public Health. 2021;18:8224. 34360515 10.3390/ijerph18158224 PMC8346172

[12] Berlin KS Parra GR Williams NA. An introduction to latent variable mixture modeling (part 2): longitudinal latent class growth analysis and growth mixture models. J Pediatr Psychol. 2014;39:188–203. 24277770 10.1093/jpepsy/jst085

[13] Galloway M Barlow P Jordan J Lo E. Neoadjuvant chemotherapy for early breast cancer: a study on response rate and toxicity. J Clin Med. 2025;14:7362. 41156231 10.3390/jcm14207362 PMC12565014

[14] Kim R Kin T Arihiro K. Roles of tumor-infiltrating lymphocytes and antitumor immune responses as predictive and prognostic markers in patients with breast cancer receiving neoadjuvant chemotherapy. Int J Mol Sci . 2025;26:9959. 41155253 10.3390/ijms26209959 PMC12563753

[15] Wang S Liu H Wei W Zhang YL Huang L. A rare complication of thrombotic microangiopathy induced by chemotherapy for second breast cancer in a Hodgkin lymphoma survivor: a case report. AME Case Rep. 2025;9:94. 40761194 10.21037/acr-24-247 PMC12319590

[16] Chikhladze N Chelidze N Kordzaia S Zhvania M Khmaladze L. Onycholysis as a complication of taxane-based chemotherapy with concomitant cryotherapy in breast cancer patients: two case reports. Georgian Med News. 2024:109–12. 38501631

[17] Alsbrook KE Hagan Thomas T DeVito Dabbs A. Associations among self-advocacy, patient-centered communication, pain intensity, and opioid stigma in older adult female breast cancer survivors. Oncol Nurs Forum. 2025;52:168–78. 40293931 10.1188/25.ONF.168-178 PMC12056831

[18] Alsbrook KE Donovan HS Wesmiller SW Hagan Thomas T. Oncology nurses’ role in promoting patient self-advocacy. Clin J Oncol Nurs. 2022;26:239–43. 35604742 10.1188/22.CJON.239-243 PMC9202076

[19] Grujic D Giurgi-Oncu C Oprean C. Well-being, depression, and anxiety following oncoplastic breast conserving surgery versus modified radical mastectomy followed by late breast reconstruction. Int J Environ Res Public Health. 2021;18:9320. 34501926 10.3390/ijerph18179320 PMC8431465

[20] Schmidt EK Faieta J Tanner K. Scoping review of self-advocacy education interventions to improve care. OTJR (Thorofare N J). 2020;40:50–6. 31342850 10.1177/1539449219860583

[21] Schultz PV Brambila-Manso B Couto-Rosa L. Validation of Autism Stigma Knowledge - Questionnaire (ASK-Q) for Brazilian Portuguese. Explor Res Clin Soc Pharm. 2024;15:100495. 39290418 10.1016/j.rcsop.2024.100495 PMC11405970

[22] Stopic V Jost ST Haupt J. Validation study of the Parkinson’s Disease Stigma Questionnaire (PDStigmaQuest). J Parkinsons Dis. 2024;14:1469–80. 39331110 10.3233/JPD-240224 PMC11492143

[23] Park S Seo K. Validity and reliability of the Korean Version of the Weight Self-Stigma Questionnaire (WSSQ-K). Nurs Rep. 2023;13:835–43. 37368340 10.3390/nursrep13020073 PMC10304408

[24] Sexton A Gargan B Taylor J Bogwitz M Winship I. Living with hereditary haemorrhagic telangiectasia: stigma, coping with unpredictable symptoms, and self-advocacy. Psychol Health. 2019;34:1141–60. 30931645 10.1080/08870446.2019.1583341

[25] Shaban M Mohammed HH Gomaa Mohamed Amer F Shaban MM Abdel-Aziz HR Ibrahim AM. Exploring the nurse-patient relationship in caring for the health priorities of older adults: qualitative study. BMC Nurs. 2024;23:480. 39010101 10.1186/s12912-024-02099-1 PMC11247866

[26] Curran MJ Gannon R Rivera RR Li Y Fitzpatrick JJ. Facilitators of and barriers to the therapeutic nurse-patient relationship: perceptions from psychiatric mental health nurses. J Am Psychiatr Nurses Assoc. 2025;31:176–82. 38910436 10.1177/10783903241257633

[27] Hong Y Chen M Chen C Qiu M. Abusive supervision and nursing students’ intention to leave the nursing profession: a moderated mediation model of emotional exhaustion and the nurse-patient relationship. BMC Nurs. 2024;23:361. 38816748 10.1186/s12912-024-02025-5 PMC11137899

[28] Aznar-Huerta A Moreno-Poyato AR Cardo-Vila G Vives-Abril T Leyva-Moral JM. Understanding the process of acceptance within the nurse-patient therapeutic relationship in mental health care: a grounded theory. Healthcare (Basel). 2024;12:2233. 39595431 10.3390/healthcare12222233 PMC11593617

[29] Feng Y Liu C Tao S. Developing and validating the nurse-patient relationship scale (NPRS) in China. BMC Nurs. 2024;23:255. 38649929 10.1186/s12912-024-01941-w PMC11034141

[30] Sheppard FH Livsey KR Martin JD. Nurse-led goal setting activities to enhance older adult health care and self-advocacy. Int J Older People Nurs. 2025;20:e70043. 40765153 10.1111/opn.70043 PMC12326115

[31] Ruth-Sahd LA Mann M Cawood ER. Innovative nurse externship that fosters interprofessional collaboration, resilience, and self-advocacy. Nursing. 2022;52:15–9. 10.1097/01.NURSE.0000872436.71363.6c 36129499

